# AI-mediated translation presents two possible futures for academic publishing in a multilingual world

**DOI:** 10.1371/journal.pbio.3003215

**Published:** 2025-06-23

**Authors:** Tatsuya Amano, Lynne Bowker, Andrew Burton-Jones

**Affiliations:** 1 School of the Environment, The University of Queensland, Brisbane, Queensland, Australia; 2 Centre for Biodiversity and Conservation Science, The University of Queensland, Brisbane, Queensland, Australia; 3 Département de langues, linguistique et traduction, Université Laval, Québec City, Québec, Canada; 4 School of Business, The University of Queensland, Brisbane, Queensland, Australia

## Abstract

As the availability and performance of AI for language editing and translation continues to improve, we can imagine a future in which everyone can use their own language to write, assess and read science. The question is, how can we achieve it?

## Teaser

As the availability and performance of artificial intelligence for language editing and translation continues to improve, we can imagine a future in which everyone can use their own language to write, assess, and read science. The question is, how can we achieve it?

In an ideal world, academic publishing is about “*removing barriers and promoting inclusion in knowledge creation and sharing, and publishing research outputs that enable everyone to learn from, reuse and build upon scientific knowledge*”. The use of English as the common language of science has boosted international scholarly communication, including publishing, but has also posed unignorable barriers to the progress and application of science. For example, reading, writing, and publishing papers requires much more time and effort for scientists whose first language is not English compared to native English speakers [1], which can lead to higher levels of anxiety and dissatisfaction [2]. Centralizing the publication of research around English also undermines the ability of people with limited English proficiency to read and use the research [3] and drives international research to ignore science published in other languages [4]. The scientific community urgently needs to move beyond the use of English as the singular default language to ensure that all scientists (and other actors and stakeholders) have an equal opportunity to access, contribute to, and benefit from science, regardless of their backgrounds [5].

A primary reasons that science has not yet become fully multilingual is that translation can be slow and costly. Artificial intelligence (AI), however, may finally allow us to overcome this problem, as it can provide useful, often free or affordable, support in language editing and translation [6]. Large language models are already widely used in academic writing, especially in countries where English is not widely spoken [7]. Existing AI has limitations, most notably variations in the availability and performance of AI among different languages [8]. However, assuming that this situation will continue to improve, we can now imagine two futures for academic publishing in which we could leverage the power of AI to overcome language barriers and improve equity in the publication, synthesis, and application of science.

**Fig 1:**
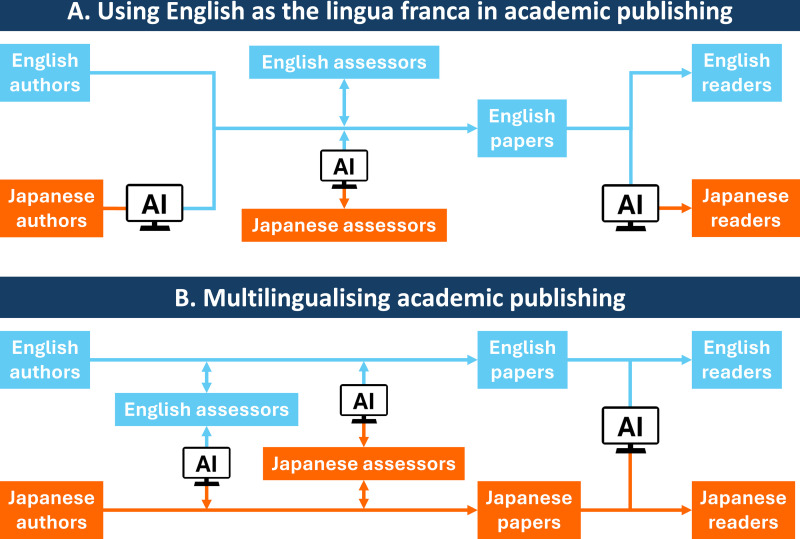
Two futures for academic publishing using artificial intelligence language tools. Information communicated in English is shown in pale blue and that in a language other than English (Japanese in this example) is shown in orange. **(A)** In Future 1, scientific papers continue to be published in English. Artificial intelligence (AI) is used by those with limited English proficiency to translate information between their preferred language and English when writing, assessing (reviewing and editing) and reading papers. **(B)** In Future 2, scientific papers are published in any language of the authors’ choice (English or Japanese in this example). AI is used by those without proficiency in the publication language (e.g., Japanese) to translate information between that language and their preferred language (e.g., English). Here, only one non-English language is shown for simplicity, but translation may be between different non-English languages. For example, Reviewer #1 may read a Japanese-written paper and provide feedback in Spanish, Reviewer #2 may do the same in Chinese, the Editor may read the paper and the reviewers’ comments and provide feedback in Arabic, and the authors would read all of their feedback in Japanese, all through AI translation.

In Future 1, English would continue to be the lingua franca in science (Fig 1A). Although international journals would continue to publish in English, researchers with limited English proficiency could write papers in their own language and use AI to translate them into English before submission. They could also use AI to translate English-language papers into their own language when reading, reviewing, and editing those papers. Scientific knowledge would continue to be centralized around English, but the use of AI would help to make science more easily producible and accessible for those with limited English proficiency.

This future is less ‘disruptive’ because the scientific community would continue to operate using the existing publishing system. Also, as most AI models are disproportionately trained on English-language data, translation to and from English tends to be of higher quality than translation between non-English languages. However, Future 1 would have various drawbacks. Inequality between fluent and non-fluent English speakers would remain; any negative consequences of using AI in academic publishing, including translation inaccuracies and the financial cost of using AI tools, would be imposed only on non-fluent English speakers. As long as scientific knowledge is centralized around English, the ongoing ‘domain loss’ (the idea that the growing use of English in a certain domain leads to other languages losing status and eventually not being used at all [9]) for other languages will not decelerate and could even intensify. New concepts in science may be described only in English, and other languages may not even have terms for new concepts. People will not be able to talk about science in their own language easily, and this may further isolate science from non-scientists, potentially leading to a lower uptake of science in decision-making and less trust in science among the general public.

Now imagine another future, Future 2, in which academic journals publish papers written in any language (Fig 1B). This would enable authors to write and submit papers in their own language. Here, assessors (editors and reviewers) and recipients of science (both scientists and non-scientists) would use AI to read those papers in their own language. A major advantage of this future would be that everyone can use their own language for science, which would help maintain and promote the diversity of science and scientific languages. This would be a giant leap forwards for 95% of the world’s population (native speakers of languages other than English) who, at present, have little choice but to conduct science in English. Publishing science in other languages could also help to halt domain loss and facilitate the understanding and use of science in countries where English is not widely spoken.

That said, making academic publishing multilingual in a fair way will not be easy. For example, even with AI tools, will people find and read English-language papers and papers in unfamiliar languages equally frequently? Will the evaluation of papers written in a non-English language be conducted in an unbiased manner? Given that AI translation is inevitably imperfect, especially for low-resource languages, this future could introduce another bias in the assessment, visibility and use of science depending on the language of publication. Various pragmatic roadblocks also exist; for instance, literature search systems would need to integrate multilingual metadata and cross-language information retrieval to allow users to search for literature written in different languages. The AI-driven automation of literature searching, screening and data extraction using multilingual models should help researchers to better use evidence in multiple languages [10]. However, encouraging publishing in languages beyond English will require a systemic change, as the current English-based assessment of science and scientists drives scientists to publish in English, even in countries where English is not widely spoken.

We should be mindful about the inaccuracy of AI translation and its consequences in science. But we also need to understand trade-offs between the consequences of AI inaccuracy and the considerable benefits of overcoming existing language barriers. The acceptable risk of using AI translation likely differs depending on purpose and discipline. Training subject area experts continues to be essential for spotting improbable AI translations, and the involvement of such experts will be necessary, especially where misunderstanding evidence can have serious implications.

Despite these and other counterarguments, Future 2 is still our preferred future, as it would truly democratize academic publishing. People often express concern that AI translation does not meet a phantom gold standard. But the reality is that issues around inaccurate use and understanding of English are already widespread in every process of academic publishing, and these are now entirely attributed to a lack of effort by researchers with lower English proficiency. The use of AI can at least create a more level playing field in the sense that it does not privilege one language above all others. There is no single big step towards Future 2; what we need are small stepping stones, such as experimenting with multilingual publication in just a few languages. To make a start, we have launched various initiatives [11] and encourage others to follow suit. AI will no doubt be integrated into all elements of the academic publishing workflow in the near future, and we believe now is the time for the scientific community to start discussing how we can use its benefits to move towards making science multilingual.

## Abbreviation

AI: artificial intelligence
